# Cloning, Expression and Effects of *P. americana* Thymosin on Wound Healing

**DOI:** 10.3390/ijms20194932

**Published:** 2019-10-05

**Authors:** Jie Jing, Xiaohong Sun, Chuang Zhou, Yifan Zhang, Yongmei Shen, Xiaomao Zeng, Bisong Yue, Xiuyue Zhang

**Affiliations:** 1 Key Laboratory of Bio-Resources and Eco-Environment of Ministry of Education, College of Life Sciences, Sichuan University, Chengdu 610065, China; jingjie321@126.com (J.J.); bsyue@scu.edu.cn (B.Y.); 2 Sichuan Key Laboratory of Medicinal American Cockroach, Good doctor Pharmaceutical Group, Chengdu 610000, China; 3 Department of Herpetology, Chengdu Institute of biology, Chinese Academy of Sciences, Chengdu 610041, China; zengxm@cib.ac.cn; 4 Sichuan Key Laboratory of Conservation Biology on Endangered Wildlife, College of Life Sciences, Sichuan University, Chengdu 610065, China

**Keywords:** *Periplaneta americana*, multimeric thymosin, prokaryotic expression, wound healing

## Abstract

The American cockroach (*Periplaneta americana*) is a medicinal insect. Its extract is used clinically to promote wound healing and tissue regeneration, but the effective medicinal components and mechanisms are not yet clear. It has been reported that human thymosin beta 4 (Tβ4) may accelerate skin wound healing, however, the role of *P. americana* thymosin (Pa-THYs) is still poorly understood. In the present study, we identify and analyze the DNA sequences of Pa-THYs by bioinformatics analysis. Then we clone, express, and purify the Pa-THYs proteins and evaluate the activity of recombinant Pa-THYs proteins by cell migration and proliferation assays in NIH/3T3 cells. To elucidate the role of Pa-THYs in wound healing, a mouse model is established, and we evaluate wound contraction, histopathological parameters, and the expressions of several key growth factors after Pa-THYs treatment. Our results showed that three THY variants were formed by skipping splicing of exons. Pa-THYs could promote fibroblast migration, but have no effect on fibroblast proliferation. In wound repair, Pa-THYs proteins could effectively promote wound healing through stimulating dermal tissue regeneration, angiogenesis, and collagen deposition. On the molecular mechanism, Pa-THYs also stimulated the expression of several key growth factors to promote wound healing. The data suggest that Pa-THYs could be a potential drug for promoting wound repair.

## 1. Introduction

Skin is the largest external organ of the human body and is vulnerable to injuries. Particularly, in elderly or diabetic patients, wound healing tends to be delayed and the risk of wound infection is increased due to vascular aging and the weakness of tissue repair ability, which may eventually lead to chronic wounds. In addition, wound treatment brings serious economic burdens and psychological pressure to society, for example, in the United States, wound treatment costs more than $30 billion a year [1]. Therefore, wound repair is one of the hot topics in the field of dermal surgery. At present, few agents have been discovered to substantially promote wound repair in patients [2]. Current strategies mainly include small molecule compounds extracted from plants or growth factors (epidermal growth factor-like proteins (EGF), and human platelet-derived growth factor (PDGF-BB). However, small molecule compounds are unstable and less active, and growth factors are expensive, which restricts their clinical application [3,4]. Therefore, the development of new drugs for wound healing has become very important.

Thymosin β4 is a small (5 kDa) peptide, containing 43 amino acids and is found in many tissues and cell lines of vertebrates, and is also known to be rich in platelets [5]. Structurally, there is only one “THY” domain in human thymosin β4 (Tβ4), which usually contains a conserved motif “LKKTET” that can form a complex with G-actin in a 1:1 ratio and inhibits G-actin to polymerize into filaments [6]. Aside from the function in actin-sequestering of Tβ4, it participates in numerous biological activities, including wound healing [7], angiogenesis [8], cardiac repair [9], anti-inflammation [10,11], hair regrowth [12], and reproduction [13]. To date, β-thymosin has been well researched in vertebrates, but there are very limited studies on invertebrates, especially in insects. With the development of bioinformatics, more and more thymosin β4 homologues from invertebrates were identified. Compared with β-thymosin in vertebrates, β-thymosin from invertebrates has more than one “THY” domain which was categorized as a multimeric β-thymosin [14]. Although their homology is relatively high, some functions are different. For example, multimeric β-thymosin can promote actin polymerization, whereas β-thymosin is thought to be a sequester protein, which suggests that the function of these two kinds of thymosin behave differently [6]. In addition, owing to exon skip splicing, many varieties of thymosin in invertebrates have several isoforms, actually, they come from the same gene and the functions of different isoforms are different [15,16]. However, most researches about multimeric β-thymosin focused on its structural characterization, the style to connect with G-actin or the expression in mRNA level, and little information has been explored on its molecular function [16,17,18].

*P. americana*, is a traditional Chinese medicine. Its extract has a good effect on wound healing [19]. Yet the key effective medicinal composition is still unknown, which hinders the further clinical utilization and exploitation of *P. americana*. Considering that both vertebrate Tβ4 and the extract of *P. americana* have good effects on wound healing, it is surprising that there are no reports about *P. americana* thymosin. Therefore, the aim of this study is to get insights into the role of *P. americana* thymosin in wound healing using animal models. Herein, we obtained the DNA sequences of thymosin β4 homologue from *P. americana* genome and transcriptome by bioinformatics analysis. By in vitro and in vivo experiments, we evaluate the function of Pa-THYs in wound healing. The present study first demonstrates the role of multimeric β-thymosin protein for promoting wound healing in animal models and provides a potential drug for wound healing.

## 2. Results

### 2.1. Bioinformatics Analysis of P. americana Thymosin

**Figure 1:**
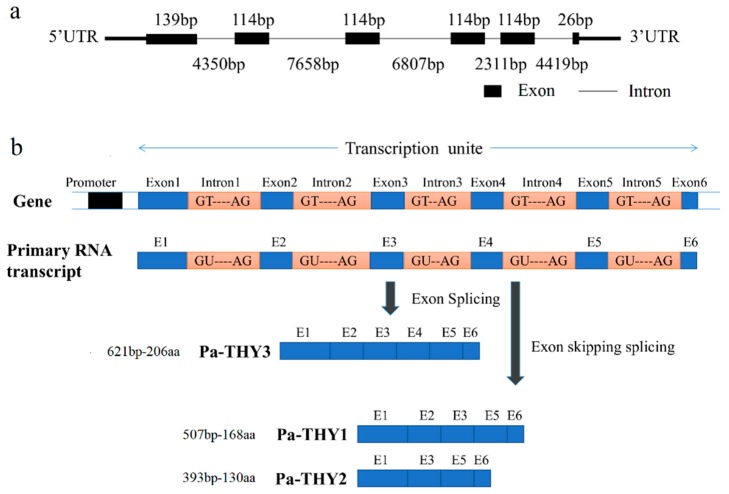
The gene structure of β-thymosin in *P. americana*. (**a**) The structure and composition of β-thymosin in *P. americana*. (**b**) The exon skipping splicing process of *P. americana* thymosin (Pa-THYs).

Based on the genome and transcriptome databases of *P. americana* which were established by our laboratory, we identified the genome and transcript sequences of Pa-THYs by bioinformatics analysis. There are one genome sequence and three transcript sequences of Pa-THYs. The genome sequence, which contained six exons: exon 1 (139 bp), exon 2 (114 bp), exon 3 (114 bp), exon 4 (114 bp), exon 5 (114 bp), and exon 6 (26 bp) and five introns: intron 1 (4350 bp), intron 2 (7658 bp), intron 3 (6807 bp), intron 4 (2311 bp), and intron 5 (4419 bp) (Figure 1a).

Due to skipping splicing of exons, three different transcripts were formed, which named as THY1, THY2 and THY3. THY3 contains all the exon sequences, while exon 4 is absent in THY1 and exon 2 and 4 are absent in THY2 (Figure 1b). These three transcript sequences were deposited in GenBank. They contain 507, 393, and 621 base pairs and encode 168, 130, and 206 amino acids respectively. The molecular weight of the THYs was 19 kDa, 14.6 kDa, and 23.4 kDa, and PI (isoelectric point) was 6.15, 5.70, and 5.95, respectively. The corresponding proteins were named as Pa-THY1, Pa-THY2, and Pa-THY3. No signal peptide was detected in Pa-THYs by the SignalP 3.0 software. These three proteins are hydrophilic non-transmembrane proteins. The secondary structure of the protein is mainly composed of an alpha helix and irregular curl. According to sequence BLAST (Basic Local Alignment Search Tool) results, the longest sequence of THY3 was wholly contained THY1 and THY2. By using motif scan software, we found that THY3 have five “THY” domains, THY1 and THY2 have four and three “THY” domains, respectively (Table 1).

### 2.2. Function Domain Analysis of P. americana Thymosin

**Figure 2:**
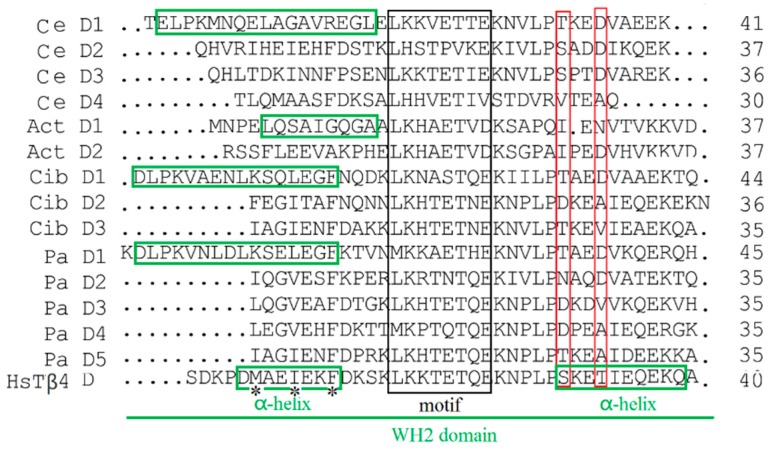
Function domains analysis of thymosins. Alignment of five Pa-THYs domains with the other four thymosins from different species (Ciboulot (Cib D, three), Actobidin (Act D, two), tetra thymosinβ (Ce D, four), and human thymosin β4 (HsTβ4 D, one)). α-helix, motif sequences, and function residues were marked by green, black and red rectangle, respectively.

According to the results of sequence alignment, Pa-THY isoforms 1, 2, and 3 resembled the “assembly-promoting form” like other multimeric thymosin, which can promote a free barbed-end filament elongation [6]. This is because they have a long N-terminal helix (green rectangle) and unstable C-terminal structure which was decided by two function residues (red rectangle) [20,21] (Figure 2). Additionally, multimeric thymosin contains more than two “THY” domains, which can bind at least two G-actins [17]. The “THY” domain has a highly conserved region. In vertebrates, the conserved motif sequence is LKKTET [13], while in *P. americana* and other invertebrates the motif sequences are LKH(R)TET, MKKAET, and MKPTQT (black rectangle), respectively (Figure 2). Pa-THY isoforms 1, 2, and 3 have 4, 3, and 5 domains, respectively, which means that they can combine more and different numbers of G-actin, hence their properties may be different from each other.

### 2.3. Expression and Purification of Recombinant Protein Pa-THYs

**Figure 3:**
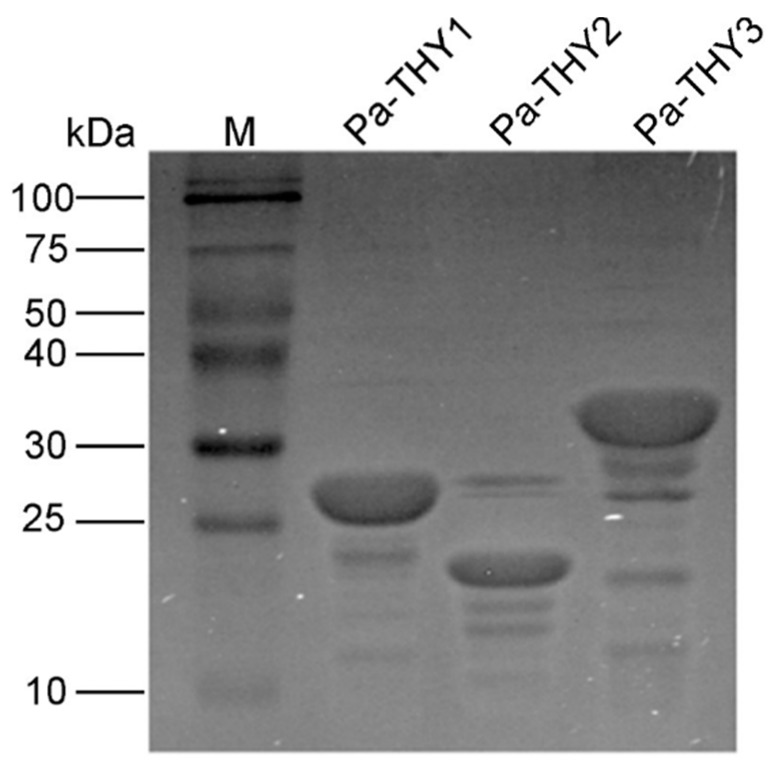
The purification of recombinant protein Pa-THYs: lane 1, marker (M) proteins and their corresponding molecular masses; lane 2, the concentrated Pa-THY1 protein; lane 3, the concentrated Pa-THY2 protein; lane 4, the concentrated Pa-THY3 protein.

Recombinant plasmids (PET-THYs) were transformed into *E coli* BL21 (DE3) (Novagen, USA). The results were confirmed by bacterial PCR and Sanger sequencing (TSINGKE, Beijing). Then, the *E. coli* was induced to express the THYs protein and Ni-affinity chromatography was used to purify the proteins. As expected, after being analyzed by SDS-PAGE, recombinant protein Pa-THY1, Pa-THY2 and Pa-THY3 with a molecular mass of approximately 25 kDa, 20 kDa and 30 kDa were detected (Figure 3). All of the above proteins were soluble. In order to get higher concentration protein, the Millipore was used to concentrate these proteins. After that, the concentration of protein was about 3 μg/μL (Pa-THY1), 2 μg/μL (Pa-THY2), and 3 μg/μL (Pa-THY3), respectively.

### 2.4. Pa-THYs Promoted Cell Migration of Fibroblasts

**Figure 4:**
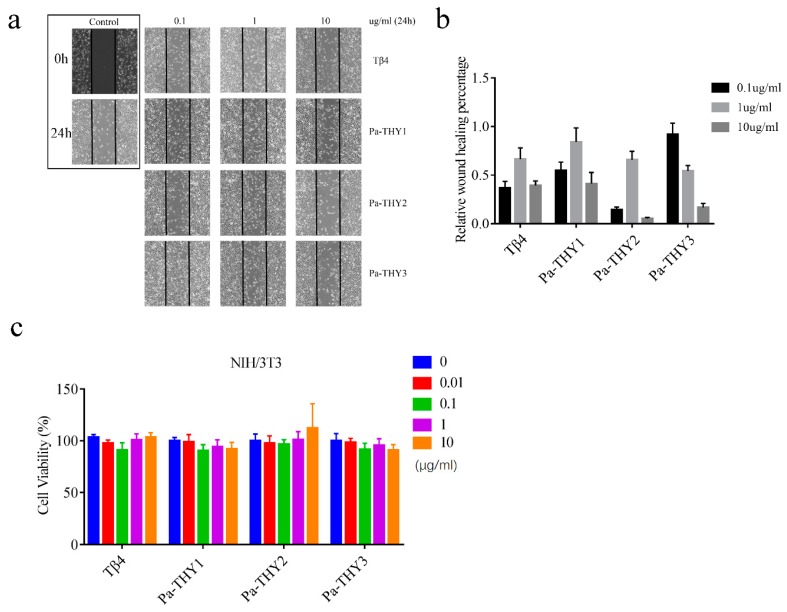
Effects of Pa-THYs on the migration and proliferation of fibroblasts. (**a**) Representative images of NIH/3T3 cells treated by Tβ4 and Pa-THYs proteins (0, 0.1 μg/mL, 1 μg/mL and 10 μg/mL). Magnification, ×200. (**b**) Quantification of wound-healing assays in NIH/3T3 cells treated with Tβ4 and Pa-THYs proteins. The wound healing percentage was compared with control group. (**c**) Cell viability of NIH/3T3 cells treated by Tβ4 and Pa-THYs proteins with different concentrations (0, 0.01 μg/mL, 0.1 μg/mL, 1 μg/mL, and 10 μg/mL).

To confirm the bioactivity and the ability for wound healing of purified proteins (Pa-THYs), the fibroblasts (NIH/3T3) were used to evaluate the ability of Pa-THYs on cell migration and proliferation. An in vitro cell scratch was performed to investigate the effect of Pa-THYs on the migration of fibroblasts. The results showed that Pa-THYs proteins promoted migration of fibroblasts with different concentrations. Based on the quantitative analysis of cell migration rates, Pa-THYs and Tβ4 obviously accelerated the migration of fibroblasts compared to PBS (phosphate buffer saline), and Pa-THY3 had a stronger effect than others during cell migration in a low concentration (0.1 μg/mL). For Tβ4, Pa-THY1 and Pa-THY2, the best concentration for cell migration was 1 μg/mL, while for Pa-THY3 was 0.1 μg/mL. Increasing protein concentration revealed a negative effect for cell migration in Tβ4 and all Pa-THYs proteins (Figure 4a,b).

MTT (3-(4,5-dimethyl-2-thiazolyl)-2,5-diphenyl-2-H-tetrazolium bromide, Thiazolyl Blue Tetrazolium Bromide) assays were employed to determine the effects of Pa-THYs on the proliferation of fibroblasts. Compared to the control group, all Pa-THYs and Tβ4 with different concentrations had no effect on the proliferation of fibroblasts (Figure 4c).

### 2.5. Recombinant Protein Pa-THYs Promoted Wound Healing

**Figure 5:**
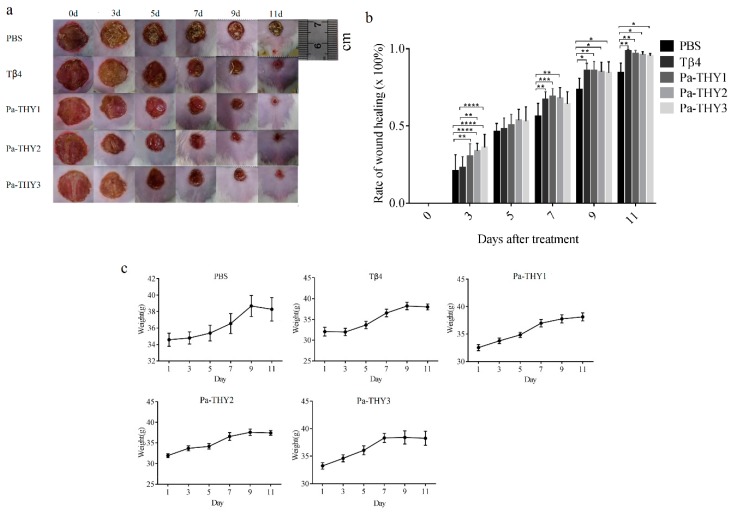
Effects of Pa-THYs proteins on wound healing in mouse models. (**a**) Representative images of skin wounds treated by Tβ4 and Pa-THYs proteins at indicated time points (0 d, 3 d, 5 d, 7 d, 9 d, and 11 d). (**b**) Quantification data of wound-healing closure in mice treated with Tβ4 and Pa-THYs proteins at indicated time points (0 d, 3 d, 5 d, 7 d, 9 d, and 11 d). * *p* < 0.05; ** *p* < 0.01; *** *p* < 0.001; **** *p* < 0.0001. (**c**) The body weight of mice treated with Tβ4 and Pa-THYs proteins at indicated time points (0 d, 3 d, 5 d, 7 d, 9 d, and 11 d).

Previous data showed considerable migration effects of Pa-THYs on fibroblasts and initially proved that Pa-THYs were involved in wound healing. To observe the effect of Pa-THYs on wound healing, a full-thickness skin wound model was established on the dorsal region of mice. Mice were treated with Pa-THYs proteins every day (24 h interval), with PBS treatment as a negative control and Tβ4 treatment as a positive control. The body weight and area of wounds were measured every two days. The surface changes of dermal wounded skin and the images of the wound appearance were observed and obtained every two days after treatment. During the wound healing process, all wounds were dry and had a large scar at first, then the scar of Tβ4 and Pa-THYs groups became moist and granulation tissue appeared while those in the PBS group were about to fall off (Figure 5a). Compared to PBS and Tβ4 groups, Pa-THYs groups showed obvious wound contraction since day 3. Based on the statistical data, Pa-THYs groups significantly accelerated wound healing compared with the PBS group at day 3, Pa-THYs and Tβ4 treatment significantly accelerated wound healing compared with PBS group at day 9 and 11 (Figure 5b). The results also showed that the mice’s body weight was not affected after treatment (Figure 5c). Obvious abnormal behavior or noticeable toxicity were not observed.

### 2.6. Pa-THYs Promoted Wound Healing by Accelerating Dermal Regeneration

**Figure 6:**
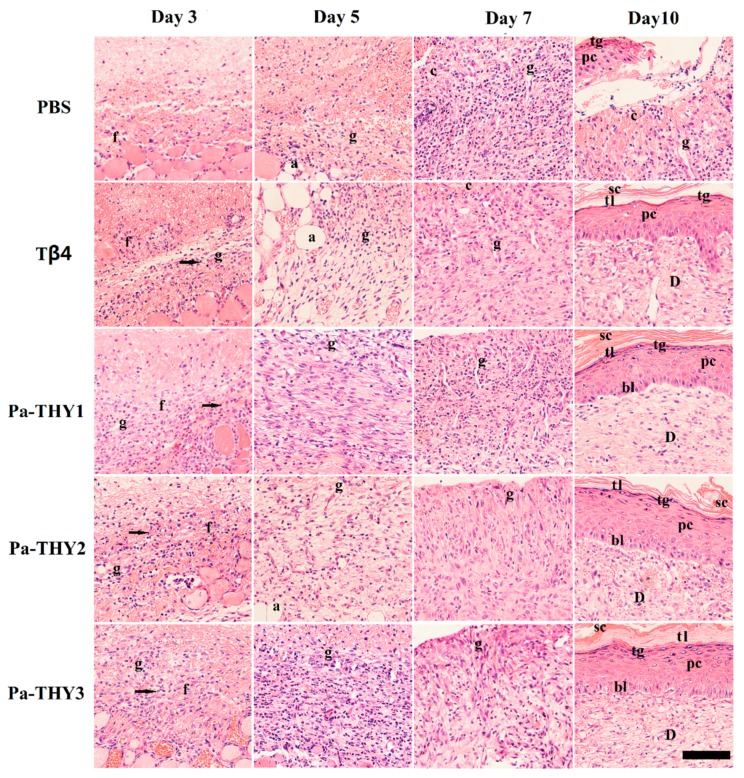
The H&E (hematoxylin-eosin) staining of dermal tissues treated with Tβ4 and Pa-THYs proteins. a: adipose tissue; c: connective tissue; f: fibroblasts; g: granulation tissue; sc: stratumcorneum; tl: transparent layer; tg: granular layer; pc: prickle cell layer; bl: basal layer; D: dermis; monocytes: marked by black arrowhead (Scale bar = 100 μm, ×200).

To examine the effect of Pa-THYs on dermal regeneration, we collected the dermal tissues of mice treated by Tβ4 and Pa-THYs proteins at indicated time points (3 d, 5 d, 7 d, and 10 d). We analyzed the formation of granulation tissue and the proliferation of fibroblasts and inflammatory cells by H&E (hematoxylin-eosin) staining. Compared with the PBS group, our results indicated that there were a large number of fibroblasts and monocytes that migrated to the bottom of the wound area in Tβ4 and Pa-THYs treated groups on day 3 and few granulations were formed. On day 5, compared with the PBS group, there were a large number of granulation tissues were formed in Pa-THYs and Tβ4 treated groups. On day 7, as expected, the results clearly demonstrated that a high level of inflammation reaction happened in the PBS group. By contrast, the inflammatory response was milder in Tβ4 and Pa-THYs treated groups, and granulation tissues almost filled the whole wound area. On day 10, except for the PBS group, inflammatory cells nearly disappeared in the other treated groups and the wound area displayed better epithelialization, forming a complete epithelial structure (stratum corneum, hyaline layer, granular layer, spinous cell layer, basal layer). There was no obvious difference between the treated groups (Figure 6).

### 2.7. Pa-THYs Promoted Wound Healing Through Stimulating Angiogenesis

**Figure 7:**
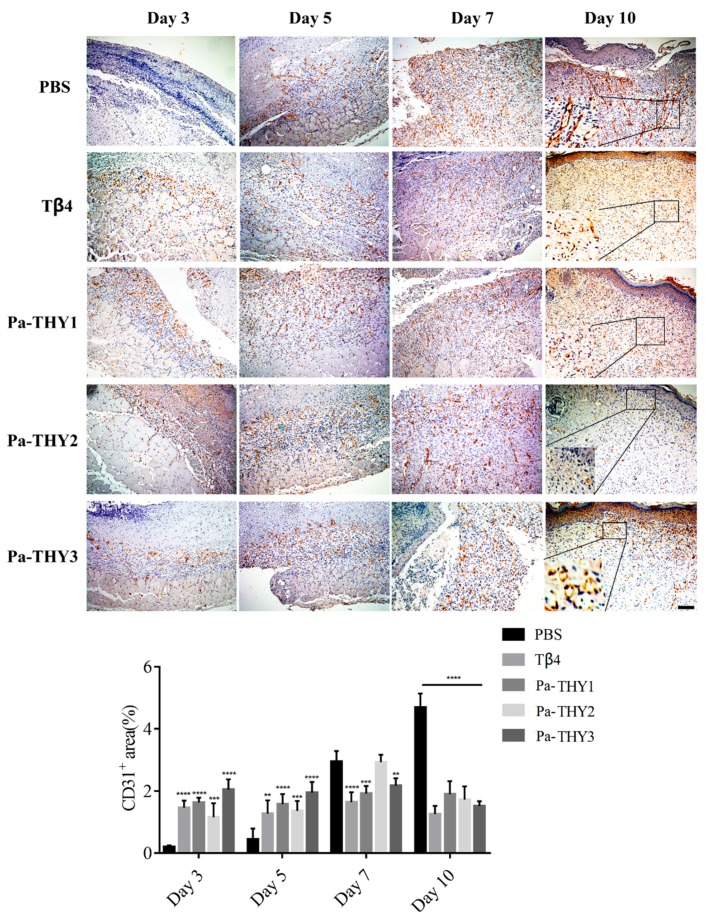
The CD31 staining of wound areas in dermal tissues treated with Tβ4 and Pa-THYs proteins (scale bar = 200 μm, ×100). All the groups compared to PBS group in certain time point, two-way ANOVA analysis,; ** *p* < 0.01; *** *p* < 0.001; **** *p* < 0.0001.

Angiogenesis is a critical process for wound healing in that newly formed blood vessels supply nutrients, amino acids, and oxygen to stimulate wound repair [22]. To evaluate the neovascularization during wound healing, the blood vessels were observed at indicated time points by CD31 (Platelet endothelial cell adhesion molecule-1) immunohistochemistry. Compared to the PBS group, Pa-THYs could significantly promote angiogenesis in the early stages of wound healing. As is seen, there were many newly formed blood vessels at the bottom of the wound area on days 3 and 5. Compared to the PBS group, the CD31^+^ area in Tβ4 and Pa-THYs treated groups were significantly increased on day 3 and day 5. After that, the new blood vessels significantly increased on day 7 and day 10 in PBS group while the new blood vessels showed no obviously variation in Tβ4 and Pa-THYs treated groups, and a large number of stripe-like blood vessels remained in the PBS group on day 10 (Figure 7).

### 2.8. Pa-THYs Promoted Wound Healing Through Stimulating Collagen Deposition

**Figure 8:**
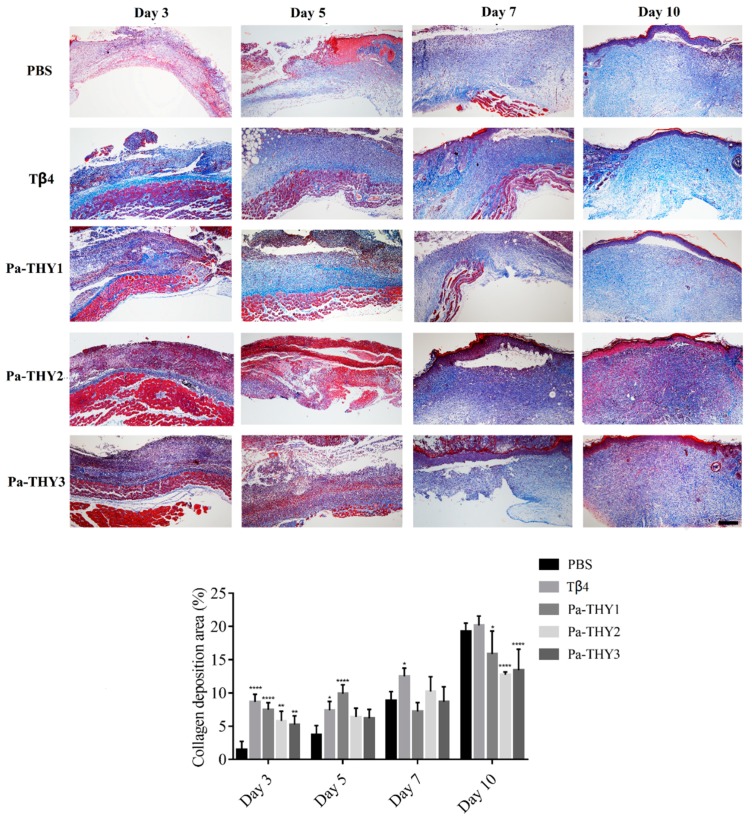
Masson staining of wound areas in dermal tissues treated with Tβ4 and Pa-THYs proteins (Scale bar = 500 μm, ×40). All the groups compared to PBS group in certain time point, two-way ANOVA analysis, * *p* < 0.05; ** *p* < 0.01; **** *p* < 0.0001.

Collagen is an important component for reconstructing dermis tissues at wound sites [23]. Masson’s trichrome staining was applied to describe collagen deposition (the blue shaded area) in dermal tissues treated with Tβ4 and Pa-THYs proteins. As showed in Figure 8, compared with the PBS group, significant accumulation of the collagen fibers was observed in the bottom of wound tissues when treated with Tβ4 and Pa-THYs proteins on day 3 and day 5. From day 5 to day 10, collagen gradually filled up with the whole wound tissue in all groups. Statistical analysis found that collagen fibers were slowly formed on day 10 in Pa-THYs groups compared to PBS and Tβ4 treated groups. These data indicated that Pa-THYs could promote wound healing through stimulating collagen deposition in early stage.

### 2.9. Pa-THYs Stimulating the Expression of Cytokines and Growth Factors

**Figure 9:**
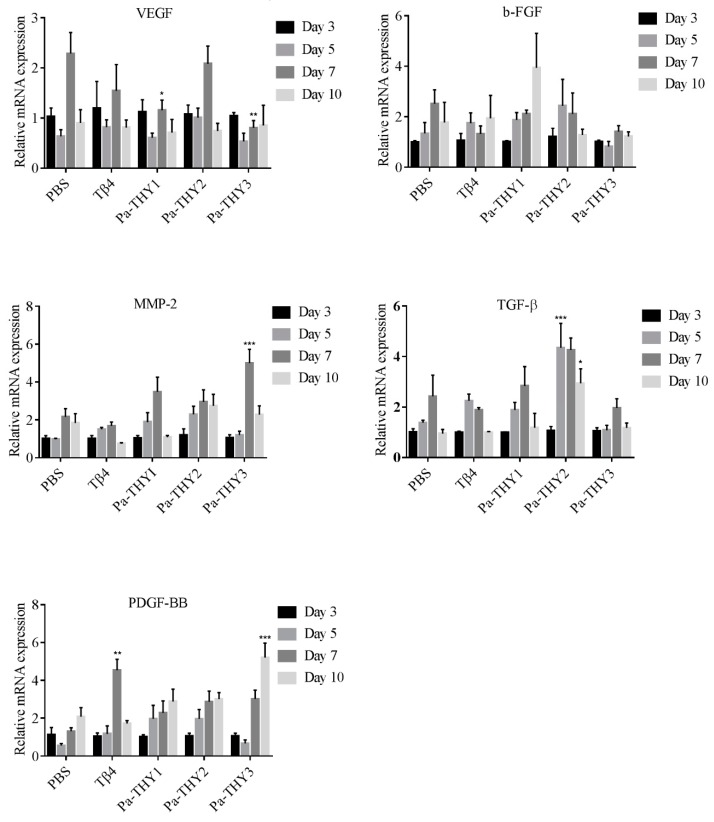
The expressions of vascular endothelial growth factor (VEGF), fibroblast growth factor (b-FGF), transforming growth factor-β (TGF-β), matrix metallopeptidase 2 (MMP-2), and platelet derived growth factor-BB (PDGF-BB) in mice treated with Tβ4 and Pa-THYs proteins were determined by qRT-PCR. All the factors were compared to PBS group in certain time point, Two-way ANOVA analysis, * *p* < 0.05; ** *p* < 0.01; *** *p* < 0.001.

To confirm the role of cytokines and growth factors to wound healing after Tβ4 and Pa-THYs proteins treatment, we examined the expression of relative factors (vascular endothelial growth factor (VEGF), fibroblast growth factor (b-FGF), transforming growth factor-β (TGF-β), matrix metallopeptidase 2 (MMP-2), and PDGF-BB at indicated time points (3 d, 5 d, 7 d, and 10 d). β-actin was used as a reference housekeeping gene to assess the different expression of factors between each group. As seen in Figure 9, our results show that all the factors participated in wound repair and that different Pa-THYs proteins may promote wound healing in different ways. Compared with the PBS group, Pa-THY1 mainly stimulated the expressions of b-FGF, MMP-2, TGF-β and PDGF-BB, but no factors significantly increased. Pa-THY2 mainly stimulated the expressions of MMP-2, TGF-β and PDGF-BB, and TGF-β significantly increased in day 5 and day 10. Pa-THY3 mainly stimulated the expressions of MMP-2 and PDGF-BB to accelerate wound healing, MMP-2 significantly increased in day 7 and PDGF-BB significantly increased in day 10.

## 3. Discussion

In vertebrates, β-thymosins usually have one conserved “THY” domain, while in invertebrates, the number of “THY” domains ranges from 2 (*Drosophila melanogaster*, NP_726909.1) to 27 (*Hydra vulgaris*, AAW82079.1) [16]. Different splicing methods could produce a variety of β-thymosin isoforms, and it can significantly increase the complexity of thymosin in invertebrates [15]. For example, there are two thymosin isoforms (HaTHY1 and HaTHY2) in *Helicoverpa armigera*, which are expressed differently in different organs and co-regulated growth and immune reaction [18]. In this study, due to exons alternative splicing, three *P. americana* thymosin isoforms were formed. The 4, 3, and 5 “THY” domains were identified in THY1, THY2 and THY3, respectively. A similar phenomenon was also found in other species such as the fruit fly [24], cotton bollworm [18], and termite [25]. Thus, the functions of these three isoforms may be different.

Multimeric thymosin could bind to more than one G-actin and enhance motility of filaments by promoting assembly in the barbed end (+). Its function was similar to profilin: G-actin complex was only occurring in multimeric thymosin [6,26]. The study found that the N-terminal a-helix amphipathic (M6, I9 and F12) and N-terminal a-helix length in β-thymosin/WH2 control the affinity of these peptides for actin, the elongation of N-terminal a-helix of β-thymosin may lead to the loss of actin sequester function [27,28]. Recent research confirmed that it is also relative to the stability of the C-terminal helix, which is mainly due to the two sites of Tβ4 (Ser31 and Thr34) were substitution [21,29]. Through sequence alignment, we found that thymosin from *P. americana* have a long N-terminal a-helix and unstable C-terminal helix. We observed that Pa-THYs not only combine more than one G-actin, but also promote G-actin assembly in the barbed end (+) of filament.

Cell migration involves dynamic change of the cytoskeleton. Multimeric β-thymosin could combine more than one G-actin to regulate assemble and disassemble of microfilaments. For example, Ciboulot (*Drosophila melanogaster*) have three “THY” domains and can bind two G-actin monomers; tetraThymosin (*Caenorhabditis elegans*) have four “THY” domains and can bind three G-actin monomers [17]. Pa-THYs could promote fibroblasts migration but have no effect on proliferation. It also demonstrated that the ability of Pa-THYs to stimulate fibroblasts migration was Pa-THY3 > Pa-THY1 > Pa-THY2. Therefore, we speculate that the different ability on cell migration may be related to the number of “THY” domains.

Wound healing is a complex physiological process of organisms, and is regulated by various cells and some intra- and intercellular signaling pathways derived from the epidermis and dermis [30]. It involves several interrelated phases, including hemostasis or coagulation (platelet aggregation and vasoconstriction), inflammation (release cytokines and remove debris), tissue regeneration (angiogenesis and granulation tissue formation) and tissue remolding (collagen deposition) [31]. Tβ4 could accelerate wound healing either directly applying to the surface of full-thickness dermal wound or giving intraperitoneal by stimulating angiogenesis, keratinocyte migration, collagen deposition and wound contraction [7,32,33,34]. It has also been found that Tβ4 have anti-inflammation properties in corneal wound healing [35], and cascade four Tβ4 (4xTβ4) was more efficient than standard Tβ4 in wound healing [36]. Researchers suggest that granulation tissue, which is mainly composed of fibroblasts, macrophages, and new blood capillaries, invades the wound space on the fourth day after injury [37]. Neovascularization occurs under hypoxic conditions, with the aim of transporting and utilizating adequate oxygen when tissue is destroyed [22]. In our research, at the early stage of wound healing, a large amount of fibroblasts, a few granulation tissues, and a few new blood vessels were observed in Pa-THYs and Tβ4 treatments. On day 7, granulation tissues were nearly filled in the whole wound, but there were still lots of inflammatory infiltrations in the PBS group. This may be one reason for delayed wound repair. Once the wound is filled up with new granulation tissue, the new blood vessels begin apoptosis [38]. On day 10, the wound in treatment groups had completely epithelialized, the inflammatory reactions and striate vessels disappeared compared with control group. Collagen is the major component for the improved strength of wound, and increasing collagen deposition can accelerate the epithelialization [39]. In this experiment, compared with the control group, the treatment groups present more collagen deposition at days 3 and 5. All of this confirmed that Pa-THYs could accelerate wound healing by promoting fibroblast migration, neovascularization, collagen deposition, and by inhibiting inflammation.

Researchers found that many growth factors are important in wound healing, such as VEGF, b-FGF, TGF-β, MMP-2, and PDGF-BB [40]. VEGF, b-FGF, and TGF-β are the most potent cytokines in promoting wound angiogenesis [41,42]. The expression of VEGF can be directly up-regulated by b-FGF, thus they have a synergistic effect [43]. MMPs were reported to be upregulated during wound healing when treated with Tβ4 [5]. PDGF-BB was approved for treatment of diabetic foot ulcers by the FDA [44], and it was reported that there is a more pronounced effect on myocardial angiogenesis when combined with PDGF-BB and b-FGF [45]. Previously, Tβ4 was reported to up-regulate the expression of VEGF and MMPs during dermal wound repair [46,47], which is in accordance with the results of our experiment. Most interestingly, researchers found that the new blood vessels were not formed after long time use Tβ4 to cure injured corneal [48]. This indicated that VEGF was not the major factor to promote angiogenesis. In this study, after treatment with protein Pa-THY1, Pa-THY2, and Pa-THY3, the expression changes of above factors were different. Pa-THY1 can obviously enhance the expression of b-FGF, MMP-2, TGF-β, and PDGF-BB. Pa-THY2 enhanced the expression of MMP-2, TGF-β, and PDGF-BB. Pa-THY3 up-regulated the expression of MMP-2 and PDGF-BB. Therefore, we suspect that different isoforms of thymosin from *P. americana* may regulate wound healing through different signal pathways. Further studies are required to evaluate the molecular mechanisms of Pa-THYs for accelerating wound healing.

## 4. Materials and Methods 

### 4.1. Bioinformatics Analysis for DNA Sequences of P. americana Thymosin

We identified the gene and transcript sequences of *P. americana* thymosin from genomic and transcriptional databases which were built by our lab [49]. The introns and exons of the *P. americana* thymosin gene sequence were analyzed by the softberry program (http://linux1.softberry.com/all.htm). According to alternative splicing, there are three different transcript variants (THY1, THY2, and THY3) in *P. americana* thymosin. All complete thymosin cDNA sequences were deposited in GenBank (accession No. MK573540, MK573541, MK573542). The DNA sequences were analyzed by the online program at NCBI (https://blast.ncbi.nlm.nih.gov/Blast.cgi). Conserved motifs were determined using Motif Scan (http://myhits.isb-sib.ch/cgi-bin/motif_scan) and signal peptide was predicted using the SignalP Server (http://www.cbs.dtu.dk/services/SignalP/). The theoretical PI and molecular mass were estimated by ExPASy (http://www.expasy.ch/tools/peptide-mass.html).

### 4.2. Cloning, Expression, and Purification of Pa-THYs

The sequences of THYs were cloned into a PET-28(a) vector, the proteins Pa-THYs were expressed in *E. coli* cells and purified by His Trap TMFF crude. Briefly, the sequences of THYs were amplified from the total cDNA of *P. americana* with specific primers, as follows: PET-Pa-F (*EcoR I*): 5′-*CGC*GAATTCATGTCGGCCCCAGTC-3′; PET-Pa-R (*Xho I*): 5′-CCGCTCGAGTTATGCTTTCTT CTCTTCATCG-3 and then cloned into PET-28(a) vector at *EcoR I* and *Xho I* recognition sites. The plasmids of PET-THY1, PET-THY2, and PET-THY3 were transformed into *E. coli* BL21 (DE3) competent cells for the final expression. The proteins (Pa-THY1, Pa-THY2, and Pa-THY3) were induced by isopropyl β-D-thiogalactoside (IPTG) at 37 °C for 6 h. The cells were harvested by centrifuging at 8000 rpm for 10 min and suspending in a 1× PBS phosphate buffer. The *E. coli* was shattered using ultrasonication, then centrifuged at 12,000× *g* for 10 min at 4 °C to remove the precipitate. The recombined proteins were purified by His Trap TMFF crude (1 mL) according to the manufacturer’s instructions and analyzed with SDS-PAGE. To get the high concentration of these proteins, they were concentrated by an ultrafiltration centrifuge tube (Millipore, Massachussettes, USA), and the total protein concentration was measured by BCA (bicinchoninic acid) protein assay kit (Biosharp, Hefei, Anhui, China) according to the manufacturer’s instructions.

### 4.3. Cell Culture

The NIH/3T3 cell line was provided by the Core Facility of West China Hospital. The NIH/3T3 cell was cultured with DMEM (HyClone, Boston, MA, USA) supplemented with 10% fetal bovine serum (FBS) at 37 °C in a humidified atmosphere of 5% CO_2_.

### 4.4. Cell Migration and Proliferation Assays 

Effects of Pa-THYs on NIH/3T3 cell migration were determined by wound healing assay. Briefly, wound healing assay was carried out in six-well plates (3 × 105 cells/well), wounds were created using a pipette tip. The cells were then rinsed with PBS to remove any free-floating cells and debris. After washing, the medium was replaced by a control medium with different concentrations (0, 0.1, 1 and 10 μg/mL) Pa-THYs, and the cells were incubated at 37 °C. The area of wound healing was observed at 0, and 24 h, and representative images for each concentration were photographed. NIH/Image J software (https://imagej.net/NIH_Image) was used for quantification of the scratch wound area based on an edge-detection and thresholding technique. We calculated the migrated area by calculating the blank area of the scratch in different time point after Pa-THYs protein treatment.

Effects of Pa-THYs on NIH/3T3 cell proliferation were determined by MTS (3-(4,5-dimethylthiazol-2-yl)-5-(3-carboxymethoxyphenyl)-2-(4-sulfophenyl)-2H-tetrazolium) (72 h) assays. Briefly, MTS assays were carried out in 96-well plates (2000 cells/well, five replicates), and the treatments of proteins were initiated at 24 h post-seeding, cells were cultured for 72 h. Then 20 μL MTS (Promega, Madison, WI, USA) was added to each well (100 μL medium) and incubated for 1–2 h at 37 °C. OD490 was measured by a Gen5 Microplate Reader (BioTek, Winooski, Vermont, USA) according to the manufacturer’s instructions. IC50 values were calculated by GraphPad Prism 5 (San Diego, CA, USA).

### 4.5. Animal Model

The *P. americana* were raised in our lab in an appropriate environment (T: 25–30 °C; humidity: 70–80%). Synthetic Tβ4 was purchased from Shanghai Zhengyan Chemical Technology Co., Ltd. (Shanghai, China). A total of 75 free of viruses, bacteria and parasites KUNMING mice (7 weeks old, male) were purchased from Da Shuo (Cheng Du, China). The mice were raised in a standard laboratory pellet diet and water ad libitum about a week to adapt the environment under controlled temperature (22–25 °C) and humidity (about 60%) with a 12 h light/ dark cycle. All animals testing used in this study were performed in accordance with guidelines of the Animal Care Committee of Sichuan University (Chengdu, China).(20190412001, 12, April 2019).

Before the mouse model was established, all mice were anesthetized by intraperitoneal injection of 10% chloral hydrate. Under sterile conditions, the dorsal hair of the mice was shaved and completely removed with hair removal cream (Veet, Tokyo, Japan), then the skin was sterilized with 75% EtOH and a circular full-thickness excisional wound on dorsal of each mouse was made, 1.2 cm in diameter, which was created by a surgical scissor. The mice were divided into five groups of fifteen animals, each including: control group with wound and PBS treatment, positive group with wound and Tβ4 (5 μg) treatment, test group I with wound and Pa-THY1 (5 μg) treatment, test group II with wound and Pa-THY2 (5 μg) treatment, test group III with wound and Pa-THY3 (5 μg) treatment. After 24 h of surgery, they were applied to the wound by pipetting the liquid (50 μL) directly into the wound area until 11 days later. The wounds were left open with no dressing.

### 4.6. Macroscopic Evaluation

Appetite and general health conditions were monitored daily, and body weight was measured every other day. For wound area study, the wound beds were photographed with a digital camera and a ruler at the specific time points (0 d, 3 d, 5 d, 7 d, 9 d, and 11 d). Wound images were used to calculate the wound area by the NIH/Image J software. The following equation was used to measure the rate of wound closure,

(1) $$y\left(n\right)=\frac{A_{0}-X_{n}}{A_{0}}\cdot 100\%$$

where y (n): the rate of wound closure (%); A_0_: wound area at day 0; X_n_: wound area at day (n). Wound healing curves were constructed by Graph Pad Prism 5.

### 4.7. Sample Collection and Histological Analysis

To examine re-epithelialization, granulation tissue, vessel counts, and collagen content of the wounds, three mice from each group were euthanized at 3, 5, 7, and 10 days after treatment. The entire wound and adjacent wound were harvested down to the fascia, and then bisected through the center of the lesion to get the largest diameter of the wound, one-part tissue was stored in liquid nitrogen to detect the expression of relative factors and another part was fixed in formalin solution (4%) for histological evaluation of wound healing. The fixed tissue samples were routinely processed and embedded in paraffin, 4 μm sections of middle wound bed were stained with hematoxylin and eosin (HE), CD31 antibody and Masson Trichrome. The positive area of CD31 and collagen deposition were measured by NIH/Image J software (https://imagej.net/NIH_Image).

### 4.8. RNA Extraction and qRT-PCR

Total RNA was extracted frozen tissues of wound healing models by using Trizol reagent (Takara, Japan) according to the manufacturer’s instructions. The purity and concentration of total RNA were determined by UV Spectrophotometer (Eppendorf, Hamburg, Germany) and 1% agarose gel electrophoresis. The first-stand cDNA was synthesized by the HiScript® II 1st Strand cDNA Synthesis Kit (Vazyme, Nanjing, China) following the manufacturer’s instructions. The primers of the quantitative real-time PCR (qRT-PCR) was designed by Prim5 software (Table 2). The qRT-PCR was used to detect relative expression of b-FGF, PDGF-BB, TGF-β, VEGF, and MMP-2 by an ABI 7500 real-time PCR detection system (ABI, Carlsbad, CA, USA), and the relative expression of these genes were normalized by an internal control (β-actin). The qRT-PCR final reaction volume was 20 μL which was added according to the instructions, and under the following conditions: 2 min of pre-denaturation at 95 °C, followed by the 30 cycles for 15 s, 59–60 °C for 15 s, and 72 °C for 30 s, and each reaction was performed in triplicate. Relative expression of genes was calculated using the 2^−ΔΔCT^ method.

### 4.9. Statistical Analysis

Data was shown as mean ± SD. The data were performed using a student’s t-test (for two groups), one-way ANOVA followed by Tukey’s test (for more than two groups) and two-way ANOVA followed by multiple comparisons were performed using Graph Pad Prism 5 (San Diego, CA, USA). The *p*-value < 0.05 was considered to be significant, the *p*-value < 0.01 was considered to be extremely significant.

## Figures and Tables

**Table 1:** Results of bioinformatics analysis of Pa-THYs.

	Pa-THY1	Pa-THY2	Pa-THY3
Amino acid	168	130	206
Molecular weight (kDa)	19,039.55	14,582.52	23,435.50
Isoelectric point (PI)	6.15	5.70	5.95
Signal peptide	none	none	none
Transmembrane	no	no	no
Hydrophilic/Hydrophobic	Hydrophilic	Hydrophilic	Hydrophilic
Subcellular locations	plasma membrane and nucleus	plasma membrane and nucleus	plasma membrane and nucleus
Alpha helix (%)	52.38%	49.23%	51.%
Random coil (%)	45.24%	47.69%	42.72%
Beta turn (%)	2.38%	3.08%	3.40%
Extended strand (%)	0%	0%	2.43%
“THY” domains	4	3	5

**Table 2:** Oligonucleotide primers used in the experiments.

Primer Name	Sequence F (5′–3′)	Sequence R (5′–3′)
VEGF	F:CTACTGCCGTCCGATTGA	R:TCTCCGCTCTGAACAAGG
TGF-β	F:AATACGTCAGACATTCGGGAAGCA	R:GTCAATGTACAGCTGCCGTACACA
b-FGF	F:TGCTTCCACCTCGTCTGTCT	R:GAGGCAAAGTGAAAGGGACC
MMP-2	F:GAACTTGCGATTATGCCATGATGAC	R:TCTGAGGGATGCCATCAAAGAC
PDGF-BB	F:CCAGGACGGTCATTTACG	R:TGGTCTGGGTTCAGGTTG
β-actin	F:CATCCGTAAAGATCTATGCCAAC	R:ATGGAGCCACCGATCCACA

## Abbreviations

IPTG	isopropyl β-D-thiogalactoside
Tβ4	human thymosin β4
THY	*P. americana* thymosin
VEGF	vascular endothelial growth factor
TGF-β	transforming growth factor-β
b-FGF	fibroblast growth factor
MMP-2	matrix metallopeptidase 2
PDGF-BB	platelet derived growth factor-BB
